# First Discovery of Two Polyketide Synthase Genes for Mitorubrinic Acid and Mitorubrinol Yellow Pigment Biosynthesis and Implications in Virulence of *Penicillium marneffei*

**DOI:** 10.1371/journal.pntd.0001871

**Published:** 2012-10-18

**Authors:** Patrick C. Y. Woo, Ching-Wan Lam, Emily W. T. Tam, Chris K. F. Leung, Samson S. Y. Wong, Susanna K. P. Lau, Kwok-Yung Yuen

**Affiliations:** 1 State Key Laboratory of Emerging Infectious Diseases, The University of Hong Kong, Hong Kong; 2 Research Centre of Infection and Immunology, The University of Hong Kong, Hong Kong; 3 Carol Yu Centre for Infection, The University of Hong Kong, Hong Kong; 4 Department of Microbiology, The University of Hong Kong, Hong Kong; 5 Department of Pathology, The University of Hong Kong, Hong Kong; University of Tennessee, United States of America

## Abstract

**Background:**

The genome of *P. marneffei*, the most important thermal dimorphic fungus causing respiratory, skin and systemic mycosis in China and Southeast Asia, possesses 23 polyketide synthase (PKS) genes and 2 polyketide synthase nonribosomal peptide synthase hybrid (PKS-NRPS) genes, which is of high diversity compared to other thermal dimorphic pathogenic fungi. We hypothesized that the yellow pigment in the mold form of *P. marneffei* could also be synthesized by one or more PKS genes.

**Methodology/Principal Findings:**

All 23 PKS and 2 PKS-NRPS genes of *P. marneffei* were systematically knocked down. A loss of the yellow pigment was observed in the mold form of the *pks11* knockdown, *pks12* knockdown and *pks11pks12* double knockdown mutants. Sequence analysis showed that PKS11 and PKS12 are fungal non-reducing PKSs. Ultra high performance liquid chromatography-photodiode array detector/electrospray ionization-quadruple time of flight-mass spectrometry (MS) and MS/MS analysis of the culture filtrates of wild type *P. marneffei* and the *pks11* knockdown, *pks12* knockdown and *pks11pks12* double knockdown mutants showed that the yellow pigment is composed of mitorubrinic acid and mitorubrinol. The survival of mice challenged with the *pks11* knockdown, *pks12* knockdown and *pks11pks12* double knockdown mutants was significantly better than those challenged with wild type *P. marneffei* (P<0.05). There was also statistically significant decrease in survival of *pks11* knockdown, *pks12* knockdown and *pks11pks12* double knockdown mutants compared to wild type *P. marneffei* in both J774 and THP1 macrophages (P<0.05).

**Conclusions/Significance:**

The yellow pigment of the mold form of *P. marneffei* is composed of mitorubrinol and mitorubrinic acid. This represents the first discovery of PKS genes responsible for mitorubrinol and mitorubrinic acid biosynthesis. *pks12* and *pks11* are probably responsible for sequential use in the biosynthesis of mitorubrinol and mitorubrinic acid. Mitorubrinol and mitorubrinic acid are virulence factors of *P. marneffei* by improving its intracellular survival in macrophages.

## Author Summary

*Penicillium marneffei* is the most important thermal dimorphic fungus causing respiratory, skin and systemic mycosis in China and Southeast Asia. Its genome possesses a large number of polyketide synthase (PKS) genes, which should be responsible for synthesis of secondary metabolites such as pigments, antibiotics and mycotoxins. Using *state-of-the-art* gene knockdown and ultra high performance liquid chromatography-photodiode array detector/electrospray ionization-quadruple time of flight-mass spectrometry technologies, we discovered that the yellow pigment of *P. marneffei* was composed of mitorubrinol and mitorubrinic acid and was synthesized by two PKS genes, named *pks12* and *pks11*. This represents the first discovery of PKS genes responsible for mitorubrinol and mitorubrinic acid biosynthesis, in which *pks12* and *pks11* are probably responsible for sequential use in the biosynthesis of mitorubrinol and mitorubrinic acid. Using a mouse model and human and mouse macrophage cell line models for *P. marneffei* infection, we also discovered that mitorubrinol and mitorubrinic acid are virulence factors of *P. marneffei* by improving its intracellular survival in macrophages.

## Introduction

*Penicillium marneffei* is the most important thermal dimorphic fungus causing respiratory, skin and systemic mycosis in China and Southeast Asia [1], [2], [3], [4]. After the discovery of *P. marneffei* in 1956, only 18 cases of human diseases were reported until 1985 [5]. The appearance of the HIV pandemic, especially in China and Southeast Asian countries, saw the emergence of the infection as an important opportunistic mycosis in HIV positive patients. About 8% of AIDS patients in Hong Kong are infected with *P. marneffei* [6]. In northern Thailand, penicilliosis is the third most common indicator disease of AIDS, following tuberculosis and cryptococcosis [2]. Besides HIV positive patients, *P. marneffei* infections have been reported in other immunocompromised patients, such as transplant recipients, patients with systemic lupus erythematosus and those on corticosteroid therapy [7], [8], [9], [10].

Polyketides are a diverse group of secondary metabolites produced by microorganisms. Some of the best known secondary metabolites include pigments, antibiotics and mycotoxins. Polyketides are synthesized by complex enzymatic systems called polyketide synthases (PKS). In the neighborhood of the PKS genes also include additional genes that encode modifying enzymes forming biosynthetic clusters. The availability of more and more fungal genome sequences have enabled us to identify an unprecedented number of fungal PKS genes. However, relatively few fungal PKS genes have been definitely linked to the biosynthesis of specific polyketide secondary metabolites.

In 2002, the complete genome sequencing project of *P. marneffei* was started. At the moment, a 6× coverage of the genome has been completed [11]. Based on the genome sequence, we have assembled the complete mitochondrial genome sequence and analyzed the phylogeny, predicted the presence of a potential sexual cycle and developed a highly discriminative multilocus sequence typing scheme for *P. marneffei* [12], [13], [14]. Recently, we also reported a high diversity of PKS in the *P. marneffei* genome and characterized its melanin biosynthesis gene cluster and confirmed it as a virulence factor in *P. marneffei* [15]. Since the *P. marneffei* genome possesses 23 PKS and 2 PKS nonribosomal peptide synthase hybrid (PKS-NRPS) genes, which is of high diversity compared to other thermal dimorphic pathogenic fungi such as *Histoplasma capsulatum* (with only one PKS gene) and *Coccidioides immitis* (with 10 PKS genes), we hypothesized that the yellow pigment in the mold form of *P. marneffei* could also be synthesized by one or more PKS genes. To test this hypothesis, we systematically knocked down all the 23 PKS and 2 PKS-NRPS genes of *P. marneffei* and observed for the loss of yellow pigment in the knockdown mutants. The culture extracts of the wild type strain and the mutant strains with loss of yellow pigment were characterized using ultra high performance liquid chromatography-photodiode array detector/electrospray ionization-quadruple time of flight-mass spectrometry (UHPLC-DAD/ESI-Q-TOF-MS) to determine its chemical nature. The possible role and mechanism of the yellow pigment in virulence was also examined in a mouse model and macrophage cell line models respectively.

## Methods

### Ethics statement

The experimental protocols were approved by the Committee on the Use of Live Animals in Teaching and Research, The University of Hong Kong, in accordance with the Guidelines laid down by the NIH in the USA regarding the care and use of animals for experimental procedures.

### Strain and culture conditions

*P. marneffei* strain PM1 was obtained from an already-existing collection from the clinical microbiology laboratory in Queen Mary Hospital and the strain was anonymized. The yeast form of PM1 was used for knockdown of the PKS genes. A single colony of the fungus grown on Sabouraud dextrose agar at 37°C was inoculated into yeast peptone broth and incubated in a shaker at 37°C for 10 days.

### Knockdown of PKS genes

DNA extraction was performed using the DNeasy Plant Mini Kit according to manufacturer's instructions (Qiagen, Hilden, Germany). The extracted DNA was eluted in 50 µl of AE buffer and the resultant mixture was diluted 10× and 1 µl of the diluted extract was used for PCR.

Plasmid construction was performed according to our previous publication. For knockdown of *pks1*, plasmid pSilent-1 [16], obtained from the Fungal Genetics Stock Center, was used to construct the pPW1459 plasmid. First, the internal *pks1* fragment (sense) was amplified using primers LPW11663 5′-CCGCTCGAGTTTCAACGACCTATCGCCCACTCAA-3′ and LPW11664 5′-CCCAAGCTTGGGACAACAGCACCAAGCAGTGTGGACA-3′ (Invitrogen, USA) (Table 1). The PCR mixture (25 µl) contained *P. marneffei* DNA, PCR buffer (10 mM Tris-HCl pH 8.3, 50 mM KCl, 2 mM MgCl_2_ and 0.01% gelatin), 200 µM of each deoxynucleoside triphosphates and 1.0 U *Taq* polymerase (Applied Biosystem, USA). The mixtures were amplified in 32 cycles of 95°C for 30 s, 56°C for 30 s and 72°C for 40 s, and a final extension at 72°C for 10 min in an automated thermal cycler (Applied Biosystem, Foster City, CA, USA). The PCR product was purified using the QIAquick Gel Extraction kit (QIAgen, Germany), digested with *Xho*I and *Hin*dIII, and cloned into the *Xho*I-*Hin*dIII site of the pSilent-1 plasmid, resulting in pPW1459-1. Second, the internal *pks1* fragment (antisense) was amplified with primers LPW11665 5′-GGGGTACCTTTCAACGACCTATCGCCCACTCAA-3′ and LPW11666 5′-GAAGATCTACAACAGCACCAAGCAGTGTGGACA-3′ (Invitrogen, USA), using the PCR conditions described above. This amplified fragment was purified as described above, digested with *Bgl*II and *Kpn*I, and cloned into the *Bgl*II-*Kpn*I site of the pPW1459-1, resulting in pPW1459. The wild type *P. marneffei* strain PM1 was transformed with linearized pPW1459, using 200 µg/ml hygromycin for selection. For the other PKS genes, they were knocked down using the same protocol described above with primers listed in Table 1.

**Table 1:** Primers and plasmids used to construct polyketide knockdown mutants in present study.

Gene	Primer	RE site	F/R	Sequence (5′-3′)	Plasmid
*pks1*	LPW 11663	*Xho*I	F	CCGCTCGAGTTTCAACGACCTATCGCCCACTCAA	pPW1459
	LPW 11664	*Hind*III	R	CCCAAGCTTGGGACAACAGCACCAAGCAGTGTGGACA	
	LPW 11665	*Kpn*I	F	GGGGTACCTTTCAACGACCTATCGCCCACTCAA	
	LPW 11666	*Bgl*II	R	GAAGATCTACAACAGCACCAAGCAGTGTGGACA	
*pks2*	LPW 11381	*Xho*I	F	CCGCTCGAGATAAGCTGGTTTTGGTCGAATCGGC	pPW1320
	LPW 11382	*Hind*III	R	CCCAAGCTTGGGGGATTGAATGGTTGTTGGTCCCGAT	
	LPW 11383	*Kpn*I	F	GGGGTACCATAAGCTGGTTTTGGTCGAATCGGC	
	LPW 11384	*Bgl*II	R	GAAGATCTGGATTGAATGGTTGTTGGTCCCGAT	
*pks3*	LPW 9873	*Xho*I	F	CCGCTCGAGCCTTCTCTTTCGGATCTCTTC	pPW1294
	LPW 9874	*Kpn*I	F	GGGGTACCCCTTCTCTTTCGGATCTCTTC	
	LPW 9875	*Bgl*II	R	GAAGATCTGCCTAATGTCAAGCTTTTCG	
*pks4*	LPW 9506	*Xho*I	F	CCGCTCGAGCCAAACCACTCAGAGTAGCC	pPW1302
	LPW 9507	*Hind*III	R	CCCAAGCTTGGGACCCTGGTAGAGGAGATTCC	
	LPW 9508	*Kpn*I	F	GGGGTACCCCAAACCACTCAGAGTAGCC	
	LPW 9509	*Bgl*II	R	GAAGATCTACCCTGGTAGAGGAGATTCC	
*pks5*	LPW 11385	*Xho*I	F	CCGCTCGAGTCGACAACTCATCCAACAGATGCCA	pPW1321
	LPW 11386	*Kpn*I	F	GGGGTACCTCGACAACTCATCCAACAGATGCCA	
	LPW 11387	*Bgl*II	R	GAAGATCTTTCAACCTGAAGCTTCCGGGAGAAT	
*pks6*	LPW 11667	*Xho*I	F	CCGCTCGAGTGACCACTCAACAAAGTTCTGGGCC	pPW1460
	LPW 11668	*Kpn*I	F	GGGGTACCTGACCACTCAACAAAGTTCTGGGCC	
	LPW 11669	*Bgl*II	R	GAAGATCTCGCATTCATCGGATATGTGCAAGCT	
*pks7*	LPW 11670	*Xho*I	F	CCGCTCGAGTGCAAGTGTCACCGTATCTGGCGA	pPW1461
	LPW 11671	*Hind*III	R	CCGCTCGAGTGCAAGTGTCACCGTATCTGGCGA	
	LPW 11672	*Kpn*I	F	GGGGTACCTGCAAGTGTCACCGTATCTGGCGA	
	LPW 11673	*Bgl*II	R	GAAGATCTAGCCCCTGTCCGTGGAAAGTTGATA	
*pks8*	LPW 13722	*Xho*I	F	CCGCTCGAGGCCGCATGTGGACAACATAT	pPW1322
	LPW 13723	*Hind*III	R	CCCAAGCTTGGGTTTTGGCCCTGCTGAGCT	
	LPW 13724	*Kpn*I	F	GGGGTACCGCCGCATGTGGACAACATAT	
	LPW 13725	*Bgl*II	R	GAAGATCTTTTTGGCCCTGCTGAGCT	
*pks9*	LPW 11674	*Xho*I	F	CCGCTCGAGTGCAGGAAAGCAACTTCGGCCCTA	pPW1515
	LPW 11675	*Hind*III	R	CCCAAGCTTGGGGCATTATCACCTCGCGCAGCTCATA	
	LPW 11676	*Kpn*I	F	GGGGTACCTGCAGGAAAGCAACTTCGGCCCTA	
	LPW 11677	*Bgl*II	R	GAAGATCTGCATTATCACCTCGCGCAGCTCATA	
*pks10*	LPW 11678	*Xho*I	F	CCGCTCGAGAGAATGGCATCGACTGCCACAGGA	pPW1516
	LPW 11679	*Hind*III	R	CCCAAGCTTGGGGCCAAACTGGAAGAGCATGCGGTAT	
	LPW 11680	*Kpn*I	F	GGGGTACCAGAATGGCATCGACTGCCACAGGA	
	LPW 11681	*Bgl*II	R	GAAGATCTGCCAAACTGGAAGAGCATGCGGTAT	
*pks11*	LPW 9278	*Xho*I	F	CCGCTCGAGAAGAACCTAAGGGATTATGGAG	pPW1303
	LPW 9279	*Kpn*I	F	GGGGTACCAAGAACCTAAGGGATTATGGAG	
	LPW 9280	*Bgl*II	R	GAAGATCTGATTCAGTTCCTTTGCCAAC	
*pks12*	LPW 11824	*Xho*I	F	CCGCTCGAGTGGAATTTCACGGTTCGCAGCA	pPW1517
	LPW 11825	*Hind*III	R	CCCAAGCTTGGGTCGCCAGCAACATGTGATTCGCT	
	LPW 11826	*Kpn*I	F	GGGGTACCTGGAATTTCACGGTTCGCAGCA	
	LPW 11827	*Bgl*II	R	GAAGATCTTCGCCAGCAACATGTGATTCGCT	
*pks13*	LPW 11682	*Xho*I	F	CCGCTCGAGTAACGCTTTCGACAGGGTTGGCTTC	pPW1462
	LPW 11683	*Hind*III	R	CCCAAGCTTGGGGCAGCTCCACGATTGCAGCAATAGA	
	LPW 11684	*Kpn*I	F	GGGGTACCTAACGCTTTCGACAGGGTTGGCTTC	
	LPW 11685	*Bgl*II	R	GAAGATCTGCAGCTCCACGATTGCAGCAATAGA	
*pks14*	LPW 11686	*Xho*I	F	CCGCTCGAGTTATCAAATGCTCGCAGTACGGGCC	pPW1477
	LPW 11687	*Kpn*I	F	GGGGTACCTTATCAAATGCTCGCAGTACGGGCC	
	LPW 11688	*Bgl*II	R	GAAGATCTGCGTTGTGCAAAAGAGCCAAGCTT	
*pks15*	LPW 9879	*Xho*I	F	CCGCTCGAGTTGACGTGAACAATACTTCC	pPW1295
	LPW 9880	*Kpn*I	F	GGGGTACCTTGACGTGAACAATACTTCC	
	LPW 9881	*Bgl*II	R	GAAGATCTTGTGTCGAGACTCAAGCT	
*pks16*	LPW 9882	*Xho*I	F	CCGCTCGAGCTTAGGAGAGGCGAATAAGAAG	pPW1296
	LPW 9883	*Kpn*I	F	GGGGTACCCTTAGGAGAGGCGAATAAGAAG	
	LPW 9884	*Bgl*II	R	GAAGATCTTGGCTATCTGCACAAGCT	
*pks17*	LPW 9885	*Xho*I	F	CCGCTCGAGGCGGGATATCACAATGCA	pPW1518
	LPW 9886	*Kpn*I	F	GGGGTACCGCGGGATATCACAATGCA	
	LPW 9887	*Bgl*II	R	GAAGATCTTTCGTGAACCAAGAAGCC	
	LPW 9888	*Hind*III	R	CCCAAGCTTGGGTTCGTGAACCAAGAAGCC	
*pks18*	LPW 11689	*Xho*I	F	CCGCTCGAGCAAATCGTCTTATCAGAGGGACTGC	pPW1463
	LPW 11690	*Kpn*I	F	GGGGTACCCAAATCGTCTTATCAGAGGGACTGC	
	LPW 11691	*Bgl*II	R	GAAGATCTGGAAGACCACCGATTGTGCAAGCT	
*pks19*	LPW 11692	*Xho*I	F	CCGCTCGAGTTTCATGGACCAAATCTCTCGCCG	pPW1478
	LPW 11693	*Hind*III	R	CCCAAGCTTGGGCCGTTACGGTTGATGCTCTCCATGA	
	LPW 11694	*Kpn*I	F	GGGGTACCTTTCATGGACCAAATCTCTCGCCG	
	LPW 11695	*Bgl*II	R	GAAGATCTCCGTTACGGTTGATGCTCTCCATGA	
*pks20*	LPW 11696	*Xho*I	F	CCGCTCGAGTTGAGGCGTATTATGACCCTTCGGG	pPW1323
	LPW 11697	*Kpn*I	F	GGGGTACCTTGAGGCGTATTATGACCCTTCGGG	
	LPW 11698	*Bgl*II	R	GAAGATCTAACGTCTTGCATCCTCCTGTTGGG	
*pks21*	LPW 12317	*Xho*I	F	CCGCTCGAGAAGACAGAACTGTGCCGGGTTGATG	pPW1519
	LPW 12318	*Hind*III	R	CCCAAGCTTGGGTGAATGATTTCGCAGACTGCTGTCG	
	LPW 12319	*Kpn*I	F	GGGGTACCAAGACAGAACTGTGCCGGGTTGATG	
	LPW 12320	*Bgl*II	R	GAAGATCTTGAATGATTTCGCAGACTGCTGTCG	
*pks22*	LPW 11703	*Xho*I	F	CCGCTCGAGATCGCAAGCTCATCGCCAAACG	pPW1479
	LPW 11704	*Hind*III	R	CCCAAGCTTGGGTGGCCATTACGCCACAACTGGACT	
	LPW 11705	*Kpn*I	F	GGGGTACCATCGCAAGCTCATCGCCAAACG	
	LPW 11706	*Bgl*II	R	GAAGATCTTGGCCATTACGCCACAACTGGACT	
*pks23*	LPW 11828	*Xho*I	F	CCGCTCGAGGTCGGATTGAACTTTCGTGACGTCG	pPW1562
	LPW 11829	*Hind*III	R	CCCAAGCTTGGGATCTGCGTAATGCTTCCCCAGCCA	
	LPW 11830	*Kpn*I	F	GGGGTACCGTCGGATTGAACTTTCGTGACGTCG	
	LPW 11831	*Bgl*II	R	GAAGATCTATCTGCGTAATGCTTCCCCAGCCA	
*pks24*	LPW 11991	*Xho*I	F	CCGCTCGAGATAACGCTTGGACAGAGCACTG	pPW1563
	LPW 11992	*Hind*III	R	CCCAAGCTTGGGCCTTTCCTGGTTGGGTCTCA	
	LPW 11993	*Kpn*I	F	GGGGTACCATAACGCTTGGACAGAGCACTG	
	LPW 11994	*Bgl*II	R	GAAGATCTCCTTTCCTGGTTGGGTCTCA	
*pks25*	LPW 12638	*Xho*I	F	CCGCTCGAGAGATGAAGACCTTGGCGGCTT	pPW1564
	LPW 12639	*Hind*III	R	CCCAAGCTTGGGTCACCACGAGCATAGCCATTGG	
	LPW 12640	*Kpn*I	F	GGGGTACCAGATGAAGACCTTGGCGGCTT	
	LPW 12641	*Bgl*II	R	GAAGATCTTCACCACGAGCATAGCCATTGG	

To construct the *pks11pks12* double knockdown mutant, the *pks11* fragment (sense) was amplified using primers LPW20378 5′-CCGCTCGAGGTATCAACACGGAAACCGACAA-3′ and LPW20379 5′-TGGCATGTGTGGTTGGTCTGCCATTCTGCGTTATCGGTAGAA-3′. The *pks12* fragment (sense) was amplified using primers LPW20380 5′- CAGACCAACCACACATGCCA-3′ and LPW20381 5′- CCCAAGCTTGGGCAGGACAAGTCTCACTGCTATTGA-3′. The two PCR products were used as template for fusion PCR to construct the *pks11pks12* fragment (sense) by using primers LPW20378 and LPW20381. The *pks11pks12* fragment (antisense) was also amplified by the same method by replacing LPW20378 with LPW20382 5′- GGGGTACCGTATCAACACGGAAACCGACAA-3′ and LPW20381 with LPW20383 5′-GAAGATCTCAGGACAAGTCTCACTGCTATTGA-3′. The sense and antisense *pks11pks12* fragments were cloned into pSilent-1 as described above.

### Real-time quantitative RT-PCR

Total RNA was extracted using RiboPure-Yeast (Ambion, USA). The RNA was eluted in 70 µl of RNase-free water and was used as the template for real-time RT-PCR. Reverse transcription was performed using the SuperScript III kit (Invitrogen, USA). Real-time RT-PCR assays was performed as described previously [17], for *pks1* fragment with primers LPW11663 and LPW11664 (Table 1), using actin with primers LPW8614 5′-CAYACYTTCTACAAYGARCTCC-3′ and LPW8615 5′-KGCVARRATRGAACCACC-3′ for normalization. cDNA was amplified in a LightCycler 2.0 (Roche, Switzerland) with 20 µl reaction mixtures containing FastStart DNA Master SYBR Green I Mix reagent kit (Roche, Switzerland), 2 µl cDNA, 2 mM MgCl_2_ and 0.5 mM primers at 95°C for 10 min followed by 50 cycles of 95°C for 10 s, 57°C (55°C for actin gene) for 5 s and 72°C for 23 s (36 s for actin gene). For the other PKS genes, real-time RT-PCR was performed using the protocol described above with primers listed in Table 1.

### Sequence and phylogenetic analysis of PKS11 and PKS12

Introns were predicted by performing pairwise alignment with the annotated *Talaromyces stipitatus* (teleomorph of *Penicillium emmonsii*) complete genome sequence. Domains of PKS11 and PKS12 were predicted using the Conserved Domains Database of NCBI and PFAM (http://pfam.sanger.ac.uk/search?tab=searchSequenceBlock) and manual inspection of multiple alignments of PKS11 and PKS12 and their homologous sequences.

### Extraction of yellow pigment

Conidia of seven-day-old cultures of wild type, *pks11* knockdown, *pks12* knockdown and *pks11pks12* double knockdown mutant strains of *P. marneffei* were collected gently and immersed into 5 µl Milli-Q water. Milli-Q water without conidia was used as negative control. The mixtures were vortexed and filtered with 0.22 µm filters. Metabolic activities in the filtrates were quenched by incubating the filtrates in liquid nitrogen for 10 min. The filtrates were used for UV-Vis spectroscopic examination and UHPLC-DAD/ESI-Q-TOF-MS analysis respectively.

### UV-Vis spectroscopic analysis

The maximum absorbance of the extracts was examined by a UV-Vis spectrometer (NanoDrop 1000 spectrophotometer, Thermo scientific, USA). The absorbance was measured by using the 0.2 mm path.

### UHPLC-DAD/ESI-Q-TOF-MS analysis

For liquid chromatography, the separation was performed by a 1290 Agilent UHPLC with an Infinity DAD (Agilent Technologies, USA) and a C18 RRHD (Agilent Zorbax Eclipse Plus 2.1 mm×100 mm, 1.8 µm) column. The column temperature was maintained at 40°C. 15 µl of reconstituted sample was injected into the UHPLC instruments. The mobile buffer A consisted of 0.05% acetic acid and 5 mM ammonium acetate in water and mobile buffer B is methanol. Gradient started from 2% buffer B from 0 to 1 min, 2% to 40% buffer B from 1 to 10 min, 40% to 95% buffer B from 10 to 17 min, maintained at 95% buffer B from 17 to 20 min and equilibrated from 20 to 22 min. The flow rate was 0.35 ml/min. Separation was coupled to a 6540 Agilent Q-TOF mass spectrometer (Agilent Technologies, USA) with a jet stream ESI mode and infinity DAD. UV spectrum was collected from 200 to 640 nm with a 4-nm silt.

For MS, mass spectrometer acquisition was operated in the positive ESI mode with accurate mass ranged from 110 to 1700 m/z. Fragmentor was at 135 V and skimmer at 50 V. Drying gas flow rate was kept at 10 l/min at 300°C, the sheath gas flow was 10 l/min at 325°C, capillary voltage was 3500 V, and nebulizer was 45 psi. MS/MS acquisition was operated in the same parameter in MS acquisition. Collision energy was 5 eV for fragmentation of the targeted compounds. Mass spectrometry data were acquired at extended dynamic range with 4 spectra/s and 8 spectra/s in MS/MS mode. Mass accuracy was enhanced by automated calibrant system with two internal reference masses (121.0509 and 922.0098).

### Animal experiments

Balb/c (H-2^d^) mice (6 to 8 weeks old, 18 to 22 g) were obtained from the Laboratory Animal Unit, The University of Hong Kong [18]. The experimental protocols were approved by the Committee on the Use of Live Animals in Teaching and Research, The University of Hong Kong, in accordance with the Guidelines laid down by the NIH in the USA regarding the care and use of animals for experimental procedures. The number of animals used was kept at the minimum that still ensured statistical significance of survival differences between the experimental groups. Mice were housed in cages, under standard conditions with regulated day length, temperature and humidity, and were given pelleted food and tap water ad libitum. Ten mice were challenged intravenously with 8×10^6^ spores of wild type *P. marneffei* and another 10 mice each with *pks11* knockdown, *pks12* knockdown and *pks11pks12* double knockdown mutants respectively. Survival of the mice was recorded daily for 60 days and analyzed by Kaplan-Meier method and Log-rank test. P<0.05 was regarded as statistically significant. The experiment was performed in duplicate.

### Intracellular survival assays in J774 and THP1 macrophages

J774 macrophages (Sigma-Aldrich, USA) were grown in DMEM (Gibco, USA) supplemented with 10% fetal bovine serum and THP1 monocytes (THP1, American type culture collection, ATCC) were grown in suspension in RPMI 1640 medium (Gibco) supplemented with 10% fetal bovine serum at 37°C and 5% CO_2_.

J774 macrophages were seeded to 24-well tissue culture plates at 4×10^5^ cells per well. THP1 monocytes were seeded to 24-well tissue culture plates at 1×10^6^ cells per well and differentiated into macrophages by incubating in RPMI 1640 medium supplemented with 100 nM PMA. All cell cultures were incubated at 37°C with 5% CO_2_ for 24 h before adding fungal strains. Fresh culture media were replaced before addition of fungal strains. Infection was carried out by inoculating the conidia of wild type, *pks11* knockdown, *pks12* knockdown and *pks11pks12* double knockdown mutants of *P. marneffei* at a multiplicity of infection of 1 and incubated for 2 h to allow adhesion and invasion to occur. After 2 h, the monolayers were washed with 240 U/ml of nystatin (Sigma-Aldrich) to kill the extracellular conidia. The monolayers were then washed with warm Hank's buffered salt solution (HBSS) to remove the nystatin. Macrophages were supplemented with fresh media and incubated for 24 h. After 24 h post infection, macrophages were lysed with 1% Triton X-100 (Sigma-Aldrich) for colony forming unit (CFU) count. Cell lysates were diluted and plated on Sabouraud dextrose agar and incubated at 37°C. The CFUs recovered from cell lysates after 2 h of phagocytosis were considered as the initial inocula and were used as the baseline values for intracellular survival analysis. CFUs recovered at 24 h were used to calculate the recovery rate of fungal cells in macrophages. Experiments were repeated in triplicate to calculate the mean of intracellular survival of conidia.

### Intracellular survival assays in human neutrophils

Unpooled peripheral blood of three healthy blood donors was obtained from the Hong Kong Red Cross Blood Transfusion Service. Neutrophils were isolated according to published protocols with some modifications [19]. Human blood was diluted in HBSS without calcium and magnesium ions and undergone Ficoll-Paque density gradient centrifugation. The bottom layer which contained red blood cells (RBC) with granulocytes was collected and mixed with 3% dextran in 0.9% sodium chloride for 30 min sedimentation. Neutrophils rich supernatant at upper layer was collected and trace amount of RBC in the neutrophils layer was lysed by using RBC lysis buffer.

Conidia of wild type, *pks11* knockdown, *pks12* knockdown and *pks11pks12* double knockdown mutants of *P. marneffei* were preincubated in YPD broth for 4 h at 37°C and preopsonized by incubation in autologous human serum for 30 min at 37°C [20]. Preopsonized conidia were kept at 4°C for at least 1 h before challenge. Equal number of human neutrophils and conidia were incubated in 1 ml RPMI medium containing 10% autologous serum for 2 h. After 2 h, the cultures were washed with 240 U/ml of nystatin to kill the extracellular conidia. The cultures were then washed with HBSS. Neutrophils were supplemented with fresh media and incubated for 18 h. After 18 h post-infection, Neutrophils were lysed with 1% Igepal-CA-630 for CFU count. Cell lysates were diluted and plated on Sabouraud dextrose agar. The CFUs recovered from cell lysates after 2 h of phagocytosis were considered as the initial inocula and were used as the baseline values for intracellular survival analysis. CFUs recovered at 18 h were used to calculate the recovery rate of fungal cells in neutrophils. Experiments were repeated in triplicate to calculate the mean of intracellular survival of conidia.

### Susceptibility to hydrogen peroxide killing

Conidial suspensions of wild type, *pks11* knockdown, *pks12* knockdown and *pks11pks12* double knockdown mutants of *P. marneffei* respectively were adjusted to 4×10^3^ cells/ml in 100 mM potassium phosphate buffer (PBS) (pH 7.0) containing 25 mM hydrogen peroxide [15], [21]. At 5-min intervals, aliquots were taken, diluted in 100 mM PBS, and plated onto Sabouraud dextrose agar plates. The experiment was performed in triplicate.

### Susceptibility to ultraviolet light killing

Conidial suspensions of wild type, *pks11* knockdown, *pks12* knockdown and *pks11pks12* double knockdown mutants of *P. marneffei* respectively were adjusted to 4×10^3^ cells/ml. Appropriate dilutions of cells were plated on Sabouraud dextrose agar plates and exposed to UV light (254 nm) generated in a Crosslinker (UVP, CA, USA) at various energy settings. Percentage survival was determined by comparing the number of colonies on irradiated plates to those on non-irradiated plates [15], [21]. The experiment was performed in triplicate.

### Killing assay of antifungal peptides

Histatin 5 and PGLa were dissolved at a concentration of 1 mg/ml in 10 mM PBS, pH 7.0 respectively and stored at −20°C. Conidial suspensions of wild type, *pks11* knockdown, *pks12* knockdown and *pks11pks12* double knockdown mutants of *P. marneffei* were adjusted to 2×10^6^ cells/ml respectively in 100 mM PBS with a dilution series of peptides. After 1 h incubation, aliquots were taken, diluted in 100 mM PBS, and plated onto Sabouraud dextrose agar plates to determine viabilities. The experiment was performed in triplicate.

## Results

### *pks11* and *pks12* are responsible for yellow pigment production in *P. marneffei*

**Figure 1:**
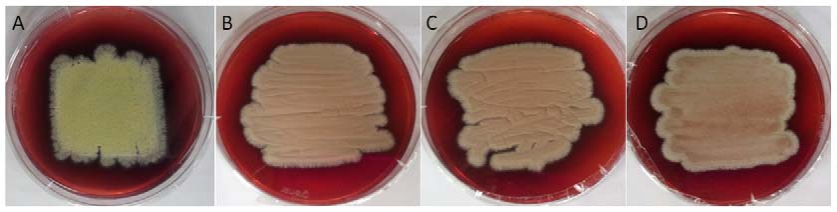
Yellow pigment production of wild type, *pks11*, *pks12* and *pks11pks12* knockdown mutants of *P. marneffei*. (A) Wild type, (B) *pks11*, (C) *pks12* and (D) *pks11pks12* knockdown mutants of *P. marneffei* were grown on Sabouraud dextrose agar after 7 days incubation at 25°C.

All 23 PKS and 2 PKS-NRPS genes of *P. marneffei* were systematically knocked down. The median transcription levels of the 25 knockdown mutants were 12.6% (range 0.1% to 40%) of that in wild type. A loss of the yellow pigment was observed exclusively in the mold form of the *pks11* and *pks12* knockdown mutants, which have *pks11* and *pks12* transcription levels 5.4% and 10.0% respectively of that in wild type (Fig. 1). A *pks11pks12* double knockdown mutant was also constructed. A loss of the yellow pigment was also observed (Fig. 1). The transcription levels of *pks11* and *pks12* were 26.5% and 9.0% respectively of that in wild type.

### Sequence and phylogenetic analysis of PKS11 and PKS12

**Figure 2:**
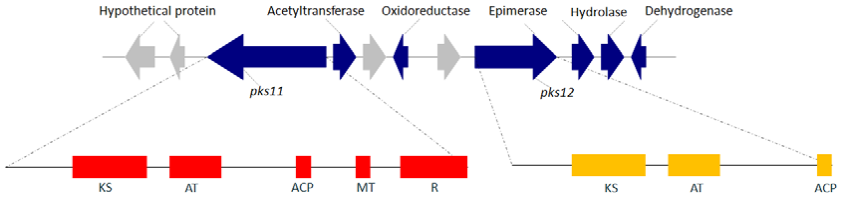
Yellow pigment biosynthesis gene cluster and domain structures of *pks11* and *pks12* in *P. marneffei*. Each arrow indicates the direction of transcription and relative sizes of the ORFs. Putative function is indicated for each gene. ACP, acyl carrier protein; AT, acyltransferase; KS, ketosynthase; MT, methyltransferase; R, thioester reductase.

The *pks11* gene is 7780 bp in length. It has one intron of 52 bp (from 607 to 658 bp). The resultant putative mRNA encodes 2575 amino acid residues with a predicted molecular mass of 282.6 kDa. The *pks12* gene is 5485 bp in length. It has one intron of 63 bp (from 439 to 501 bp). The resultant putative mRNA encodes 1806 amino acid residues with a predicted molecular mass of 197.9 kDa. PKS11 has one ketosynthase, one acyltransferase, one acyl carrier protein, one methyltransferase and one thioester reductase domains whereas PKS12 only has one ketosynthase, one acyltransferase and one acyl carrier protein domain (Fig. 2). The domain organization of PKS11 and PKS12 showed that both are fungal non-reducing PKSs.

### UV-Vis spectroscopic analysis

By UV-Vis spectroscopic analysis, an absorption maximum at 360 nm was recognized in the filtrate of wild type *P. marneffei*. This absorption maximum was not observed in the culture filtrates of the *pks11* knockdown, *pks12* knockdown or *pks11pks12* double knockdown mutants.

### UHPLC-DAD-MS analysis

**Figure 3:**
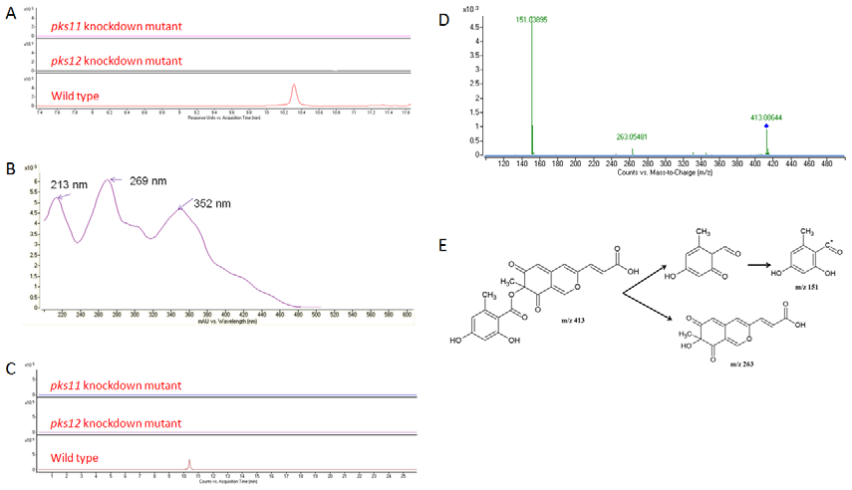
Detection of mitorubrinic acid by UHPLC-DAD/ESI-Q-TOF-MS and MS/MS analysis. (A) HPLC profiles monitored by photodiode array detector and illustrated at 360 nm, (B) UV absorption spectrum, (C) extracted ion chromatograms (m/z 413.0876), (D) MS/MS fragmentation pattern and (E) MS/MS fragmentation pathway showing the presence of mitorubrinic acid detected and identified in wild type of *P. marneffei* but not in *pks11* or *pks12* knockdown mutants.

**Figure 4:**
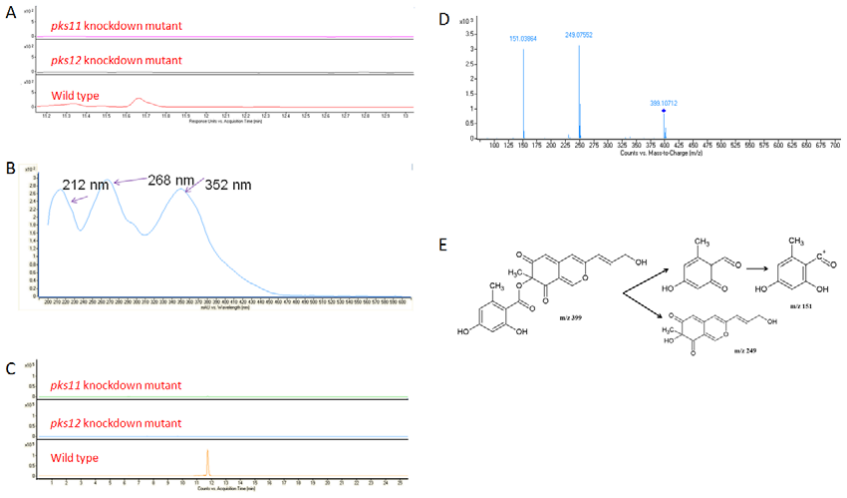
Detection of mitorubrinol by UHPLC-DAD/ESI-Q-TOF-MS and MS/MS analysis. (A) HPLC profiles monitored by photodiode array detector and illustrated at 360 nm, (B) UV absorption spectrum, (C) extracted ion chromatograms (m/z 399.10834), (D) MS/MS fragmentation pattern and (E) MS/MS fragmentation pathway showing the presence of mitorubrinol detected and identified in wild type of *P. marneffei* but not in *pks11* or *pks12* knockdown mutants.

The filtrates from wild type, *pks11* knockdown, *pks12* knockdown and *pks11pks12* double knockdown mutants of *P. marneffei* were monitored by UV-Vis spectroscopy from 200 to 640 nm and positive ion electrospray MS. To detect the presence of yellow pigment in wild type *P. marneffei* but not in the *pks11* knockdown, *pks12* knockdown or *pks11pks12* double knockdown mutants, the UHPLC profiles were monitored by DAD using UV-visible absorption at 360 nm. Peaks at 10.2 min and 11.7 min were present in wild type *P. marneffei* but not the *pks11* knockdown, *pks12* knockdown and *pks11pks12* double knockdown mutants (Fig. 3A and Fig. 4A). The two peaks were subjected to UV absorption, MS and MS/MS analyses. For the peak at 10.2 min, UV absorption analysis showed that the isolated molecule has three absorbance maxima at 213 nm, 269 nm and 352 nm respectively (Fig. 3B). MS analysis showed that this molecule has m/z of 413.0876 [M+H]^+^, which matched for C_21_H_16_O_9_ (Fig. 3C). This chemical formula was compatible with mitorubrinic acid (Fig. 3E). The fragmentation pattern of the MS/MS analysis showed a peak at m/z 151.03895, which corresponded to C_8_H_6_O_3_ and another at m/z 263.05481, which corresponded to C_13_H_10_O_6_, further confirming the isolated molecule is mitorubrinic acid (Fig. 3D and 3E). For the peak at 11.7 min, UV absorption analysis showed that the isolated molecule has three absorbance maxima at 212 nm, 268 nm and 352 nm respectively (Fig. 4B). MS analysis showed that this molecule has m/z of 399.10834 [M+H]^+^ matched for C_21_H_18_O_8_ (Fig. 4C). This chemical formula was compatible with mitorubrinol (Fig. 4E). The fragmentation pattern of the MS/MS analysis showed a peak at m/z 151.0386, which corresponded to C_8_H_6_O_3_ and another at m/z 249.0755, which corresponded to C_13_H_12_O_5_, further confirming the isolated molecule is mitorubrinol (Fig. 4D and 4E).

### Animal experiments

**Figure 5:**
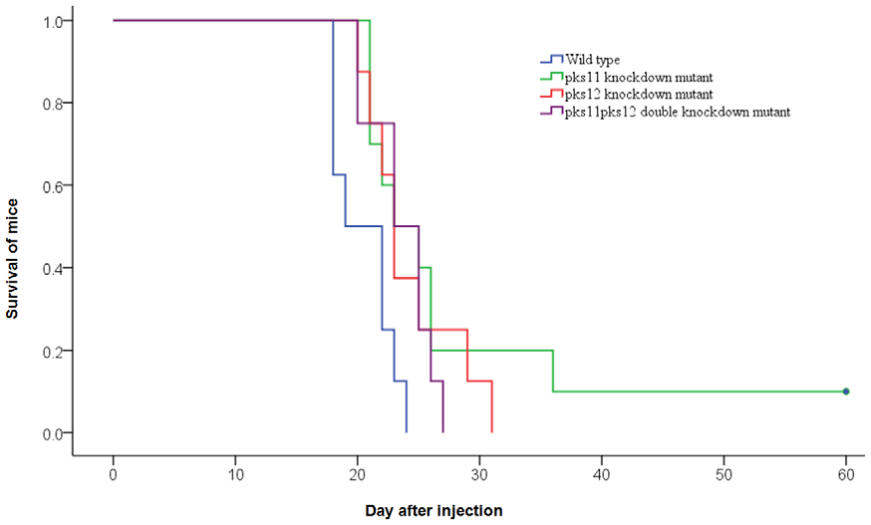
Survival of mice challenged with wild type *P. marneffei* or *pks11*/*pks12*/*pks11pks12* knockdown mutants. Groups of 10 BALB/c mice were challenged intravenously with 8×10^6^ spores. Survival was recorded daily for 60 days.

The survival of mice after intravenous challenge with wild type *P. marneffei* or the *pks11* knockdown, *pks12* knockdown and *pks11pks12* double knockdown mutants on day 60 was summarized in Fig. 5. The survival of mice challenged with the *pks11* knockdown, *pks12* knockdown and *pks11pks12* double knockdown mutants were significantly better than those challenged with wild type *P. marneffei* (P<0.05).

### Intracellular survival assays in J774 and THP1 macrophages

**Figure 6:**
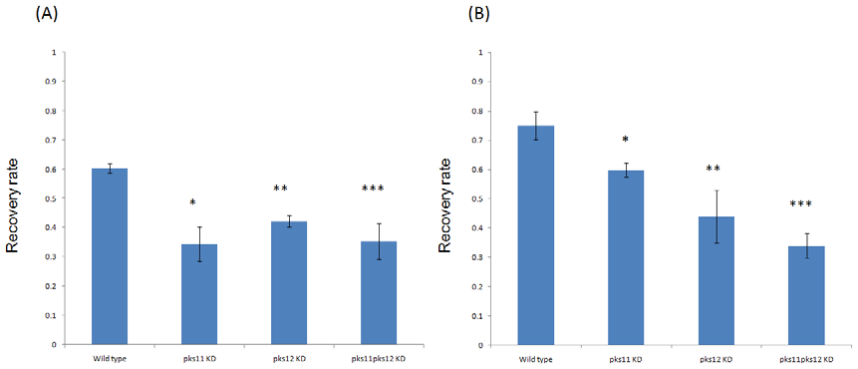
Survival of wild type *P. marneffei* and *pks11*/*pks12*/*pks11pks12* knockdown mutants in J774 and THP1 macrophages. Panels A and B represent the recovery rates of wild type, *pks11* knockdown, *pks12* knockdown and *pks11pks12* double knockdown mutants of *P. marneffei* in J774 and THP1 macrophages respectively. Error bars represent as mean ± SEM. Statistical significance between groups is indicated. *: wild type versus *pks11* knockdown mutant (p<0.05); **: wild type versus *pks12* knockdown mutant (p<0.05), ***: wild type versus *pks11pks12* double knockdown mutant (p<0.05).

The survival of wild type, *pks11* knockdown, *pks12* knockdown and *pks11pks12* double knockdown mutants of *P. marneffei* in J774 and THP1 macrophages is shown in Fig. 6. There was statistically significant decrease in survival of *pks11* knockdown, *pks12* knockdown and *pks11pks12* double knockdown mutants compared to wild type *P. marneffei* in both J774 and THP1 macrophages (P<0.05).

### Intracellular survival assays in human neutrophils

No difference was observed between the survivals of wild type, *pks11* knockdown, *pks12* knockdown and *pks11pks12* double knockdown mutants of *P. marneffei* in human neutrophils.

### Susceptibility to hydrogen peroxide killing

The relative survival of *P. marneffei* conidia capable of forming visible colonies was calculated and plotted as a function of time of incubation in 25 mM hydrogen peroxide. No difference was observed between the survivals of wild type, *pks11* knockdown, *pks12* knockdown and *pks11pks12* double knockdown mutants of *P. marneffei*.

### Susceptibility to ultraviolet light killing

No difference was observed between the survivals of wild type, *pks11* knockdown, *pks12* knockdown and *pks11pks12* double knockdown mutants of *P. marneffei* exposed to different doses of ultraviolet light.

### Killing assay of antifungal peptides

No difference was observed between the survivals of wild type, *pks11* knockdown, *pks12* knockdown and *pks11pks12* double knockdown mutants of *P. marneffei* exposed to different concentrations of histatin 5 and PGLa peptides.

## Discussion

We report the first discovery of PKS genes responsible for mitorubrinol and mitorubrinic acid biosynthesis. Mitorubrinol and mitorubrinic acid are mitorubrin derivatives, a unique subclass of azaphilones isolated from a variety of fungal species, including *Penicillium* species such as *P. rubrum* and *P. funiculosum* and *Talaromyces* (teleomorph of *Penicilllium*) species such as *T. emodensis*, *T. hachijoensis*, *T. wortmannii* var. *sublevisporus*, *T. austrocalifornicus* and *T. convolutus* [22], [23], [24]. Although it has been known that mitorubrinol and mitorubrinic acid are polyketides for decades, no PKS genes have been identified for their synthesis [22]. In this study, we systematically knocked down the 23 PKS and 2 PKS-NRPS genes in the *P. marneffei* genome and observed for loss of yellow pigment in its mold form. Two PKS genes in the same PKS gene cluster were confirmed to be responsible for biosynthesis of yellow pigment in *P. marneffei*. UV absorption, MS and MS/MS analyses all unambiguously confirmed that this yellow pigment consisted of mitorubrinol and mitorubrinic acid.

*pks12* and *pks11* are probably responsible for sequential use in the biosynthesis of mitorubrinol and mitorubrinic acid. It is well known that some polyketides, such as lovastatin and zearalenone, were synthesized by two PKSs. The first PKS serves to synthesize a starter unit for the second PKS, which possesses a starter unit ACP transacylase (SAT) domain for utilizing the advanced starter unit [25]. For zearalenone, the starter unit is a highly reduced hexaketide which encoded by a highly reducing PKS gene (*PKS13*) [26]. The starter unit is utilized by a second PKS gene (*PKS4*) which possesses SAT domain for further extension. As for mitorubrinic acid and mitorubrinol, we speculate that the first part of the biosynthesis was by PKS12, which synthesized orsellinic acid. This tetraketide is then used as a starter unit for PKS11, which possessed a putative SAT domain, in the second part of the biosynthesis. PKS11, which possessed a methyltransferase domain, also served to methylate the products, using a methyl group from S-adenosylmethionine. Interestingly, PKS12 also possessed putative SAT domain, in line with the fact that most non-reducing PKS also possess potential SAT domains irrespective of whether they require an acetate starter unit or not. Feeding experiments using isotopically or 19F labeled precursors will confirm the use of advanced starter unit for the biosynthesis of mitorubrinic acid and mitorubrinol. Notably, polyketides that are synthesized by two PKS genes commonly require a highly reducing PKS at the early stage of biosynthesis and a non-reducing PKS at the later stage, resulting in a final product consisted of a highly reducing chain and non-reducing rings. Examples include the biosynthesis of zearalenone in *Gibberella zeae* and asperfuranone in *Aspergillus nidulans* [26], [27]. However, it is rare to see a polyketide, similar to mitorubrinol and mitorubrinic acid, that is synthesized by two non-reducing PKS in a sequential manner resulting in a non-reduced polyketide product.

Mitorubrinol and mitorubrinic acid are virulence factors of *P. marneffei* by improving its intracellular survival in macrophages. Although *P. marneffei* is infecting about 8% of AIDS patients in China and Southeast Asia, the pathogenetic mechanisms of this fungus remained under studied. So far, several molecules, including superoxide dismutase and melanin, have been implicated to be associated with virulence in *P. marneffei* [15], [28]. Superoxide dismutase converts superoxide radicals into hydrogen peroxide and oxygen, whereas melanin contributed to virulence through decreased susceptibility to killing by hydrogen peroxide [15], [28]. In this study, these two PKS genes in the yellow pigment biosynthesis gene cluster responsible for mitorubrinol and mitorubrinic acid production were shown to be associated with virulence in a mouse model. However, unlike melanin, the mechanism of virulence is not related to increasing resistance to hydrogen peroxide killing and the decrease in virulence in the *pks11* knockdown, *pks12* knockdown and *pks11pks12* double knockdown mutants were also not due to decrease in survival within neutrophils, reduced resistance to antifungal peptides, decrease in growth rates or change in electron microscopic appearances of the mutants (data not shown). On the other hand, the survival of wild type *P. marneffei* was significantly better than *pks11* knockdown, *pks12* knockdown and *pks11pks12* double knockdown mutants in both murine and human macrophages. Notably, it has been shown that injection of mitorubrin to mice did not result in death of the mice, although livestock fed with feedstuff contaminated with *P. rubrum* could be poisoned, indicating that mitorubrin itself is not a toxic metabolite [22]. This is in line with the results of the present study, which suggested that the mechanism of virulence was mediated through enhancement of intracellular survival in macrophages. It is also noteworthy that the *pks11pks12* double knockdown mutant did not survive better than the *pks11* or *pks12* knockdown mutants. This is because knocking down either *pks11* or *pks12* is sufficient to abolish the pathway for mitorubrinol and mitorubrinic acid synthesis.
